# Overweight/obesity and physiological variables in workers exposed to rotating shift in a food production factory

**DOI:** 10.47626/1679-4435-2022-1005

**Published:** 2023-11-24

**Authors:** Vanessa Vallejo-Giraldo, Laura Sanchez-Medina, Elsa María Vasquez-Trespalacios

**Affiliations:** 1 Universidad CES, Epidemiologia, Medellín, Antioquia, Colombia

**Keywords:** shift work schedule, waist-hip ratio, body mass index, physiological monitoring, occupational exposure, horario de trabajo por turnos, relación cintura-cadera, índice de masa corporal, monitoreo fisiológico, exposición profesional

## Abstract

**Introduction:**

Shift work has been hypothesized as a potential risk factor for overweight/obesity or other metabolic changes. We examined the relationship between work shift and body mass index, waist-hip ratio, lipid profile, and glucose concentration in workers from a food manufacturing factory in Colombia.

**Objectives:**

To investigate the association between shift work and changes in physiological variables in food manufacturing industry workers in Medellín, Colombia.

**Methods:**

This cross-sectional study was conducted with 763 employees from a food manufacturing factory. Information was collected from the medical records from the occupational health provider institution in charge of workers’ periodic follow-up.

**Results:**

The study sample consisted of 637 (83.5%) men and 126 (16.5%) women. Mean age was 43.35 ± 9.8 years, and mean body mass index was 25.49 ± 3.23 kg/m^2^. After adjusting for potential confounders, logistic multivariate regression revealed a statistically significant association between shift work and higher body mass index and higher total cholesterol levels compared with dayshift (p < 0.05). Finally, the analysis of waist-hip ratio for each shift scheme and sex showed that this ratio was higher for rotating shift workers, with a significant difference for women.

**Conclusions:**

Significant associations were observed between shift work and overweight/obesity and hypercholesterolemia. However, these findings should be confirmed by longitudinal studies.

## Abstract (es)

**Introducción:**

El trabajo por turnos se ha asociado como un potencial factor de riesgo para sobrepeso/obesidad u otras alteraciones metabólicas. Examinamos la relación entre el trabajo por turnos y el índice de masa corporal, índice cintura-cadera, perfil lipídico y glicemia en trabajadores de la industria manufacturera de alimentos en Colombia.

**Objetivos:**

Conocer la asociación entre el trabajo por turnos y alteraciones en variables fisiológicos en trabajadores del sector de alimentos en Medellín, Colombia.

**Métodos:**

Este estudio transversal fue realizado con 763 trabajadores de una planta del sector de la industria alimentaria. La información fue analizada a partir de registros médicos en la base de datos de una institución prestadora de servicios de salud ocupacional encargada del seguimiento periódico de los trabajadores.

**Resultados:**

La muestra del estudio estuvo compuesta por 637 (83,5%) hombres y 126 (16,5%) mujeres. La edad media fue de 43,35 ± 9,8 años y el índice de masa corporal medio de 25,49 ± 3,23 kg/m^2^. Después de ajustar los posibles factores de confusión, la regresión logística multivariada reveló una asociación estadísticamente significativa entre el trabajo por turno y un mayor índice de masa corporal y nivel de colesterol total, comparado con el turno diurno (p < 0,05). Finalmente, el análisis de la relación cintura-cadera para cada esquema de turno y sexo mostro que esta medida fue mayor para los trabajadores en turno rotativo, siendo significativa la diferencia para las mujeres.

**Conclusiones:**

Se observaron asociaciones significativas entre el trabajo por turnos y el sobrepeso/obesidad e hipercolesterolemia; sin embargo, estos hallazgos deben confirmarse mediante estudios longitudinales.

## INTRODUCTION

Shift work, either in fixed or rotating schemes, is an important condition for millions of workers in Colombia and worldwide. Due to the growing social demand for 24-hour provision of goods and services, companies have been required to implement strategies to maximize productivity, including rotating shift work. Consequently, the percentage of workers in this scheme has increased over the last decades, being estimated at 20% of the worldwide population^1^ and almost 29% in Colombia, according to the 2007 first national survey on health and working conductions.^2^

An extensive literature published in the last decade suggest a possible association between shift work and changes in both workers’ physiological and psychological parameters. These effects are based on two crucial variations in their vital conditions: changes in the circadian cycle and sleep deprivation.^3^ The health consequences of these variations described so far include coronary and cerebrovascular disease, metabolic syndrome, obesity, cancer, among many others that can even lead to early death.^4-8^ Current evidence is heterogeneous and scarce in terms of our local context.^9-12^

Night workers experience a constant conflict between the pattern governed by the internal circadian cycle and the pattern imposed by the external biological rhythm, which generates an imbalance in normal body functions. The issue is even more complex in the case of rotating shift workers, because not only they are subjected to an inadequate circadian rhythm, but also the variability of their work scheme makes any adaptive attempt difficult.^4^

One of the determining factors for changes in the physiological parameters of night or rotating shift workers is lack of counterregulatory activity in cortisol secretion. Glucocorticoid secretion is under strong influence of the suprachiasmatic nucleus circadian clock, which functions as a modulator of sensitivity of soft tissues to cortisol. In normal conditions, cortisol levels reach their peak in the early morning and their nadir at midnight, in order to adjust body activities and functions to the external biological rhythm. Night or rotating shift workers develop functional hypercortisolism, with high cortisol levels in the afternoon and consequent complications resulting from metabolic syndrome. Glucocorticoids are required for the functioning of all organs and tissues, especially in respiratory, cardiovascular, immune, and musculoskeletal function; therefore, dysregulation in their levels affects multiple body system and changes several physiological parameters.^12^

The aim of the present study was to analyze the influence of fixed and rotating work schedule on blood pressure, body mass index (BMI), abdominal circumference, waist-hip ratio, lipid profile, and glucose levels. For this purpose, differences in the prevalence of overweight/obesity and/or variations of physiological parameters were compared between rotating shift workers and those with a fixed work schedule, as well as the association of these variables with other demographical and labor factors in food industry manufacture workers from the metropolitan region of Medellín.

## METHODS

### PARTICIPANTS

This cross-sectional study was conducted at a food industry factory in Medellín, Colombia.

Based on a formula for comparing proportions in independent groups and on the results of a report by Nakamura et al.,^13^ who found a percentage of obesity of 27.7% among shift workers, and of 18.8% among daytime workers, with a 95% confidence interval and a power of 80%, the recommended sample size was 357 workers. However, there was available information on 947 workers; thus, all periodic occupational health surveillance records available were assessed. A total of 947 medical records were selected from the database of an Occupational Health Services Provider Institution (Institución Prestador de Servicios de Salud, IPS) with headquarters in Medellín, Colombia, which is responsible for the periodical follow-up of the health status of workers from the factory. Individuals working in the factory for less than one year were excluded, in order to avoid the period of circadian adaptation (n = 150). Workers diagnosed with arterial hypertension, heart failure, cardio or cerebrovascular atherosclerotic disease, diabetes mellitus, or other metabolic or endocrinologic diseases before start working in the factory (n = 28) were excluded. The study also excluded workers whose more than 10% of data of interest were missed (n = 10). The custody of information was maintained through a letter of commitment and a confidentiality agreement, signed both by the researchers and the IPS. Furthermore, the present study is in compliance with Law 1,266 of 2008, which laid down the general provisions for *habeas data* and regulates the handling of information contained in personal databases.

### MEASUREMENTS

Information on demographics (age, gender, marital status, educational level, socioeconomic stratum), as well as on habits and lifestyle (smoking, alcoholism, and hours of aerobic exercise) were obtained through the occupational medical records used by the physicians from the IPS. BMI was calculated as body weight (kg), divided by height squared (m^2^). The parameters established by the World Health Organization (WHO) were applied to define the BMI classification groups.^14^ Waist circumference was measured at the midpoint between the superior border of the iliac crest and the inferior margin of the rib. WHO parameters were also applied in this case (central obesity when circumference is >88 cm in women and >102 cm in men).^15^ Hip circumference was measured at the largest circumference of buttocks. Likewise, WHO parameters were used to determine the risk threshold for cardiovascular disease (value >0.95 for men and >0.85 for women).^15^ BMI, waist circumference, and waist-hip ratio were measured by a physician during physical examination.

### STATISTICAL ANALYSIS

Participants’ characteristics were summarized for day shift workers and rotating shift workers.

Variables were expressed as mean ± standard deviation or median ± interquartile range, according to data distribution, and categorical variables as percentages. The Chi-square test and the independent t test, or the Mann-Whitney test, were used to compare means or medians between groups for normally and asymmetrically distributed data, respectively.

All data were analyzed using SPSS Statistics 21 (SPSS Inc., IBM Corp., Chicago, IL, USA).

## RESULTS

This study analyzed information on 763 workers with daytime and rotating work schedules, who were predominantly male, with a mean age of 43.35 ± 9.8 years. There were significant differences between day shift workers and rotating shift workers in terms of sex and educational level. With regard to alcohol consumption, smoking, and physical activity, no statistically significant differences were observed between the groups (p > 0.05) (Table 1).

**Table 1:** Characteristics of participant workers

Characteristics	Total (n = 763)	Day shift (n = 83)	Rotating shift (n = 680)	p-value
Sex (% male)	637 (83.5)	60 (72.3)	577 (84.9)	0.004
Age (years) (Mean ± SD)	43.35 ± 9.8	43.40 ± 9.831	43.34 ± 9.803	
Marital status (%)				
Married	450 (59)	47 (56.6)	403 (59.3)	
Single	198 (26)	24 (28.9)	174 (25.6)	
Unión Libre	75 (9.8)	8 (9.6)	67 (9.9)	
Divorced	35 (4.6)	4 (4.8)	31 (4.6)	
Widowed	5 (0.7)	0	5 (0.7)	
Educational level (%)				
Update courses	1 (0.1)	1 (0.2)	0 (0)	0.000
Master’s degree	11 (1.4)	11 (13.3)	0 (0)	
Postgraduate degree	19 (2.5)	18 (21.7)	1 (0.1)	
Elementary	33 (4.3)	31 (4.6)	2 (2.4)	
High school degree	395 (51.8)	6 (7.2)	389 (57.3)	
Incomplete high school degree	37 (4.9)	3 (3.6)	34 (5)	
Technical degree	83 (10.9)	6 (7.2)	77 (11.3)	
Technological degree	129 (16.9)	12 (14.5)	117 (17.2)	
Undergraduate degree	54 (7.1)	24 (28.9)	30 (4.4)	
Smoking				
Si	29 (3.8)	1 (1.2)	28 (4.1)	0.19
No	734 (96.2)	82 (98.8)	652 (95.9)	
Alcohol consumption				
Yes	414 (54.3)	41 (49.4)	373 (54.9)	0.346
No	349 (45.7)	42 (50.6)	307 (45.1)	
Physical activity				
Yes	574 (75.2)	59 (71.1)	515 (75.7)	0.354
No	189 (24.8)	24 (28.9)	165 (24.3)	

An analysis of physiological measurements from workers participating in the study according to their work schedule showed that the BMI of rotating shift workers is higher than that of day shift workers, representing a statistically significant difference. As for the lipid profile, higher levels of total cholesterol were found in participants working in rotating shifts compared to those working in day shifts (p < 0.05). There was no significant difference in the other blood lipid measurements between the groups (Table 2).

**Table 2:** Physiological measurements according to shift schedules

Variables	Total (n = 763)	Day shift (n = 83)	Rotating shift (n = 680)	p-value
Body mass index, kg/m^2^ (mean ± SD)	25.4 ± 3.23	24.8 ± 3.45	25.58 ± 3.1	0.038
Lipid profile (mean ± SD)				
Triglicerides	198.5 ± 122	186 ± 116	199 ± 123	0.364
Cholesterol	222 ± 49	211 ± 45	224 ± 49	0.027
LDL	138 ± 42	131 ± 35	139 ± 42	0.241
HDL	48.6 ± 13.8	47.8 ± 13.8	48.7 ± 13.9	0.599
Glucose		84.02 ± 11.7	81.78 ± 20.8	0.341
Changes in lipid profile^*^				
Yes	621 (81.4)	65 (78.3)	556 (81.8)	0.446
No	142 (18.6)	18 (21.7)	124 (18.2)	

* Changes in lipid profile without considering VLDL.

LDL = low-density lipoprotein; HDL = high-density lipoprotein; SD = standard deviation; VLDL = very low-density lipoprotein.

An analysis of the waist-hip ratio for each shift schedule and according to participant’s sex revealed that this ratio was higher among rotating shift workers compared to day shift workers, with a significant difference for female workers (Table 3).

**Table 3:** Mean waist-hip ratio stratified by sex

	Day shift	Rotating shift	p-value
Women	(n = 20) 0.73	(n = 44) 0.88	0.035
Men	(n = 61) 0.77	(n = 389) 0.88	0.561

## DISCUSSION

This study aimed to analyze the association of rotating and day work schemes with BMI, waist-hip ratio, lipid and glucose profile in a group of workers from a food industry factory located in Medellín, Colombia. In the present study, the overall prevalence of workers with overweight or obesity was 53.1%, being thus lower than the overall prevalence of overweight or obesity in adults from 18 to 64 years of age, which corresponds to 56.4% of the Colombian population, according to the 2015 Colombian National Nutrition Survey.^16^

These results may be explained by the healthy workers bias,^17^ meaning that the working population in contact with risk factor has lower morbidity and mortality rates compared with the general population, since workers are selected based on their employability status, which depends mostly on their health state before entering the labor market. Another factor to consider is that 83.5% of the sample consisted of men, who, as reported in 2015 Colombian National Nutrition Survey, have a lower prevalence of overweight or obesity compared to women.^16^

In the present study, the prevalence of overweight or obesity among rotating shift workers was 48.2%, a result close to that found among rotating shift workers from the industrial canteens of Sercoinfal CA, a company in the state Lara, Venezuela, which was 48.3%.^18^ It was also consistent with findings by Macagnan et al.^19^ showing a prevalence of overweight of 42.2% among nightshift workers in a production line in a poultry processing. Multiple studies established the role of shift role as an independent risk factor for overweight in the working population.^20-23^ The mechanism that associates shift work with higher BMI has not been completely understood yet, but different studies mention changes in circadian rhythm and sleep deprivation as triggering factors. Sleep deprivation causes a decrease in leptin levels and an increase in ghrelin levels, which seems to coincide with increased appetite and weight gain.^24^

In the sample of this study, the BMI of rotating shift workers (25.58 ± 3.1 kg/m^2^ and 24.8 ± 3 kg/m^2^, respectively) was higher than that of dayshift workers. This difference was statistically significant and is in line with findings from prospective studies, such as that of Ishizaki et al.,^22^ which also found a difference in BMI increase among rotating shift and dayshift male employees in a Japanese metal product factory after a 10-year follow-up. Similarly, Suwazono et al.^20^ reported a significantly higher increase in BMI after 14 years for rotating shift workers compared with dayshift workers in a steel company in Japan.

However, multiple studies did not find statistically significant differences in BMI among rotating shift and dayshift workers, including a local study by Vásquez-Trespalacios et al. with 200 workers in a clinical care setting, which did not reveal any statistically significant association between overweight, obesity, or waist-hip ration and shift work.^11^ In the international scenario, a study by Nakamura et al. conducted to determine whether there is an association between shift work and risk factors for coronary disease in male Japanese workers reported a mean BMI of 23 ± 3.2 kg/m^2^ for dayshift workers and of 23.2 ± 2,6 kg/m^2^ for two-shift workers.^25^

Similarly, Geliebter et al.^26^ evaluated the frequency of weight gain in rotating shift workers compared with dayshift workers and did not find significant differences in BMI between the two groups. In a systematic review of eight studies, five of which were considered to be highand three of them low-quality studies. Seven studies presented crude results for an association between shift exposure and change in body weight.

Five studies presented weight-related outcomes adjusted for potentially relevant confounders (age, sex, bodyweight at baseline, and physical activity). Consequently, the evidence for a confounders-adjusted relationship between shift work exposure and body was considered to be insufficient; therefore, the development of more and higher-quality studies about the subject was deemed necessary to further clarify the underlying mechanisms.^27^

With regard to lipid profile, higher total cholesterol levels were observed in rotating shift workers compared to dayshift workers (p < 0.05). This result is coherent with the findings from a study with male workers from a facture that manufactures semiconductors and related components in Kota Bharu, Malaysia, which reported a significantly higher prevalence of hypercholesterolemia (47.4%) and hypertriglyceridemia (42,1%) among rotating shift workers compared with dayshift workers.^28^

Vásquez-Trespalacios et al.^11^ and Knutsson^29^ found that rotating shift workers are not less likely to perform regular physical activity compared with dayshift workers, which gains relevance when it is known that this factor may potentially act as a mediator in the relationship between work shift and weight gain.^29^ However, the two studies show contrasting results for the waist-hip ratio, with the present study revealing a higher ratio among female rotating shift workers, with a statistical significant specific for women (p = 0,035).^29^

Taking only fasting glycemia as a reference, no statistically significant difference concerning glycemia was found, which is in line with findings reported by Sharma et al.,^30^ who studied 12 otherwise healthy nurses performing rotating-shift work to evaluate its effect on glucose metabolism. As previously mentioned, as with the present study, there was no difference in glucose levels between the two shift schemes. However, based on the other parameters studied, particularly on the higher postprandial glucose concentrations observed during the night shift, the authors were able to conclude that the circadian variation that occurs in the night shift could impair beta cell function.

The present study had some limitations to be considered. Firstly, although sample size is statistically relevant, there is a remarkable prevalence of the male gender in the rotating shift (>80%). Furthermore, the cross-sectional design of the study limits the analysis of a possible relationship between shift work and overweight/obesity, which prevents understanding the causality and the temporal influence of work shift exposure. Additionally, information was collected from medical records obtained in an occupational information system, where there may be discrepancies in recording the type of patient’s shift, especially when this information can be considered clinically irrelevant at the time of consultation.

Conversely, it is important to mention that some patients started to work in the day shift following a recommendation made by the occupational physician, but they had been previously exposed to rotating shift (for years, in some cases). In turn, some rotating shift workers were advised by the physician to limit night shifts as a strategy to protect their sleep, in some cases of patients with baseline epilepsy history. This was also indicated for patients who were previously healthy and developed remarkable changes in physiological variables, possibly as a strategy to prevent complications. Both scenarios did not represent a large portion of the sample population; however, these cases are described in order to acknowledge them as potential sources of information bias.

## CONCLUSIONS

The analyzed information showed significant associations between work shift and changes in physiological parameters in terms of BMI and total cholesterol for both men and women, and in terms of waist-hip ratio for women. However, these findings should be confirmed by longitudinal studies that ideally integrate the analysis of the impact of work shift on sleep, at least from the patient’s subject perspective, in order to allow for this additional correlation.

## References

[1] Santana-Herrera J, Alfano T, Escobal-Machado A. (2014). Turnos de trabajo: ¿un factor de riesgo
cardiovascular?. Med Segur Trab.

[2] Ministerio de Protección Social (2007). Primera encuesta nacional de condiciones de salud y trabajo en el
Sistema General de Riesgos Profesionales.

[3] Carrillo-Mora P, Barajas-Martínez KG, Sánchez-Vázquez I, Rangel-Caballero MF. (2018). Trastornos del sueño: ¿qué son y cuáles son
sus consecuencias?. Rev Fac Med Univ Nac Auton Mex.

[4] Ávila Darcia S (2016). Implicaciones del trabajo nocturno y/o trabajo por turnos sobre
la salud. Med Leg Costa Rica.

[5] Palacios Ruesta RC (2011). Determinación de los factores de riesgo cardiovascular en
trabajadores a turnos en plataformas marítimas de una petrolera del
Norte del Perú. Acta Med Peruana.

[6] Vyas MV, Garg AX, Iansavichus AV, Costella J, Donner A, Laugsand LE (2012). Shift work and vascular events: systematic review and
meta-analysis. BMJ.

[7] Anothaisintawee T, Reutrakul S, Van Cauter E, Thakkinstian A. (2016). Sleep disturbances compared to traditional risk factors for
diabetes development: systematic review and meta-analysis. Sleep Med Rev.

[8] Lin X, Chen W, Wei F, Ying M, Wei W, Xie X. (2015). Night-shift work increases morbidity of breast cancer and
all-cause mortality: a meta-analysis of 16 prospective cohort
studies. Sleep Med.

[9] Castillo Avila IY, Torres Llanos N, Ahumada Gomez A, Cárdenas Tapias K, Licona Castro S. (2014). Labor Stress in nursing and associate factors. Cartagena
(Colombia). Salud Uninorte.

[10] Barrera-Valencia M, Puerta-Guzmán N. (2017). Trabajar en turnos rotativos semanales no produce alteraciones en
las funciones cognitivas superiores. Rev Psicol Univ Antioq.

[11] Vásquez-Trespalacios EM, Palacio-Jaramillo V, Gómez-Parra M, Romero-Arrieta L. (2016). Shift work and work-related stress symptoms in health care
workers in a tertiary hospital in Medellín, Colombia: a
cross-sectional study. CES Psicol.

[12] Kino T, Chrousos GP. (2011). Circadian CLOCK-mediated regulation of target-tissue sensitivity
to glucocorticoids: implications for cardiometabolic
diseases. Endocr Dev.

[13] Nakamura M, Miura A, Nagahata T, Toki A, Shibata Y, Okada E (2018). Dietary intake and dinner timing among shift workers in
Japan. J Occup Health.

[14] World Health Organization (2015). Obesity and overweight.

[15] World Health Organization (2011). Waist circumference and waist-hip ratio: report of a WHO expert
consultation.

[16] Ministerio de Salud y Protección Social, Departamento
Administrativo para la Prosperidad Social, Instituto Colombiano de
Bienestar Familiar, Instituto Nacional de Salud, Universidad Nacional de
Colombia (2015). Encuesta Nacional de la Situación Nutricional ENSIN 2015.

[17] Delgado Rodríguez M, Sillero Arenas M, Gálvez Vargas R. (1994). Estudios de mortalidad proporcional: criterios de elección
de los grupos participantes. Gac Sanit.

[18] Parra D, Añez R, Vásquez D, Rojas Quintero J, Bermúdez V. (2012). Alteraciones metabólicas en empleados de una empresa de
comedores industriales de Barquisimeto, estado Lara -
Venezuela. SCEC.

[19] Macagnan J, Pattussi MP, Canuto R, Henn RL, Fassa AG, Olinto MT. (2012). Impact of nightshift work on overweight and abdominal obesity
among workers of a poultry processing plant in southern
Brazil. Chronobiol Int.

[20] Suwazono Y, Dochi M, Sakata K, Okubo Y, Oishi M, Tanaka K, Kobayashi E, Kido T, Nogawa K. (2008). A longitudinal study on the effect of shift work on weight gain
in male Japanese workers. Obesity (Silver Spring).

[21] Zhao I, Bogossian F, Turner C. (2012). A cross-sectional analysis of the association between night-only
or rotating shift work and overweight/obesity among female nurses and
midwives. J Occup Environ Med.

[22] Ishizaki M, Morikawa Y, Nakagawa H, Honda R, Kawakami N, Haratani T (2004). The influence of work characteristics on body mass index and
waist to hip ratio in Japanese employees. Ind Health.

[23] Kubo T, Oyama I, Nakamura T, Shirane K, Otsuka H, Kunimoto M (2011). Retrospective cohort study of the risk of obesity among shift
workers: findings from the Industry-based Shift Workers’ Health study,
Japan. Occup Environ Med.

[24] Taheri S, Lin L, Austin D, Young T, Mignot E. (2004). Short sleep duration is associated with reduced leptin, elevated
ghrelin, and increased body mass index. PLoS Med.

[25] Nakamura K, Shimai S, Kikuchi S, Tominaga K, Takahashi H, Tanaka M (1997). Shift work and risk factors for coronary heart disease in
Japanese blue-collar workers: serum lipids and anthropometric
characteristics. Occup Med (Lond).

[26] Geliebter A, Gluck ME, Tanowitz M, Aronoff NJ, Zammit GK. (2000). Work-shift period and weight change. Nutrition.

[27] van Drongelen A, Boot CR, Merkus SL, Smid T, van der Beek AJ. (2011). The effects of shift work on body weight change - a systematic
review of longitudinal studies. Scand J Work Environ Health.

[28] Nazri SM, Tengku MA, Winn T. (2007). Lipid disorders among male factory shift workers in Kota Bharu,
Kelantan. Med J Malaysia.

[29] Knutsson A. (2003). Health disorders of shift workers. Occup Med (Lond).

[30] Sharma A, Laurenti MC, Dalla Man C, Varghese RT, Cobelli C, Rizza RA (2017). Glucose metabolism during rotational shift-work in healthcare
workers. Diabetologia.

